# Primary dedifferentiated Liposarcoma of vagina: a first case report

**DOI:** 10.1186/s13000-020-01062-3

**Published:** 2021-01-09

**Authors:** Chuan Xie, Yangmei Shen

**Affiliations:** 1 Department of Gynecology and Obstetrics, West China Second University Hospital, Sichuan University, Chengdu, People’s Republic of China; 2 Key Laboratory of Birth Defects and Related Diseases of Women and Children, Ministry of Education, West China Second University Hospital, Sichuan University, Chengdu, Sichuan Province People’s Republic of China; 3 Department of Pathology, West China Second University Hospital, Sichuan University, Chengdu, People’s Republic of China

**Keywords:** Liposarcoma, Dedifferentiated liposarcoma, Well-differentiated liposarcoma, Vagina, Surgery, Chemotherapy

## Abstract

**Background:**

Dedifferentiated liposarcoma, one of the most deadly types of soft tissue sarcoma, is an aggressive and high-grade form of liposarcoma. Liposarcoma occurs most commonly in the retroperitoneum, extremities and trunk, but less frequently in the female genital tract. The vagina is a very rare site of origin. Herein we report the first case of dedifferentiated Liposarcoma deriving from vagina and discuss its clinical course.

**Case presentation:**

A 38-year-old female patient presented to our institution with a painless vaginal mass. Abdominal computed tomography showed a 17.6 cm× 10.4 cm solid mass originating from the right lateral wall of her vagina. Then she underwent complete surgical resection of the tumor mass, and postoperative pathological result confirmed the diagnosis of dedifferentiated liposarcoma deriving from vagina. Six courses of combination chemotherapy with pirarubicin plus ifosfamide were performed after surgery. The patient remains with no evidence of disease recurrence with 13 months of follow-up.

**Conclusions:**

Liposarcoma is very rare in female genital tract, and more rare for dedifferentiated liposarcoma in gynecologic field. Little is known about the clinical characteristics, pathological diagnosis, prognosis and optimal management strategy of vaginal dedifferentiated liposarcoma. Complete surgical resection followed by systemic chemotherapy is suggested to be standard treatment for dedifferentiated liposarcoma. Combination chemotherapy with pirarubicin and ifosfamide may be effective for treating vaginal dedifferentiated liposarcoma.

## Introduction

Liposarcoma (LPS) was firstly designated by Virchow in 1857 [1], and the term of “dedifferentiated liposarcoma” was first presented by Than Evans in 1979 [2]. The 2020 World Health Organization (WHO) classification separates liposarcoma into five histologic subtypes: well-differentiated liposarcoma (WDLPS), dedifferentiated liposarcoma (DDLPS), myxoid liposarcoma, pleomorphic liposarcoma/epithelioid liposarcoma and myxoid pleomorphic liposarcoma [3]. Each subtype of liposarcoma has unique morphology, histology, and natural history. Liposarcoma is one of the most common soft tissue sarcoma, making up nearly 20% of all malignant mesenchymal neoplasm [4]. The malignant tumor occurs most commonly in either the retroperitoneum or trunk and extremities [5]. Furthermore, the painless tumor has mesenchymal origin and tends to have multiple satellite lesions extending beyond the primary neoplasm [1].

Liposarcoma is very rare in female genital tract, but relatively common in male genitourinary system especially around the testis and spermatic cord. There have been very few reports of liposarcoma either in uterine corpus or in adnexal region [6–8]. To the best of our knowledge, there is no reported primary dedifferentiated Liposarcoma arising from vagina in English and Chinese literature up to now. Herein we report the first known case of dedifferentiated Liposarcoma deriving from vagina, and discuss its clinical course.

## Case report

**Fig. 1:**
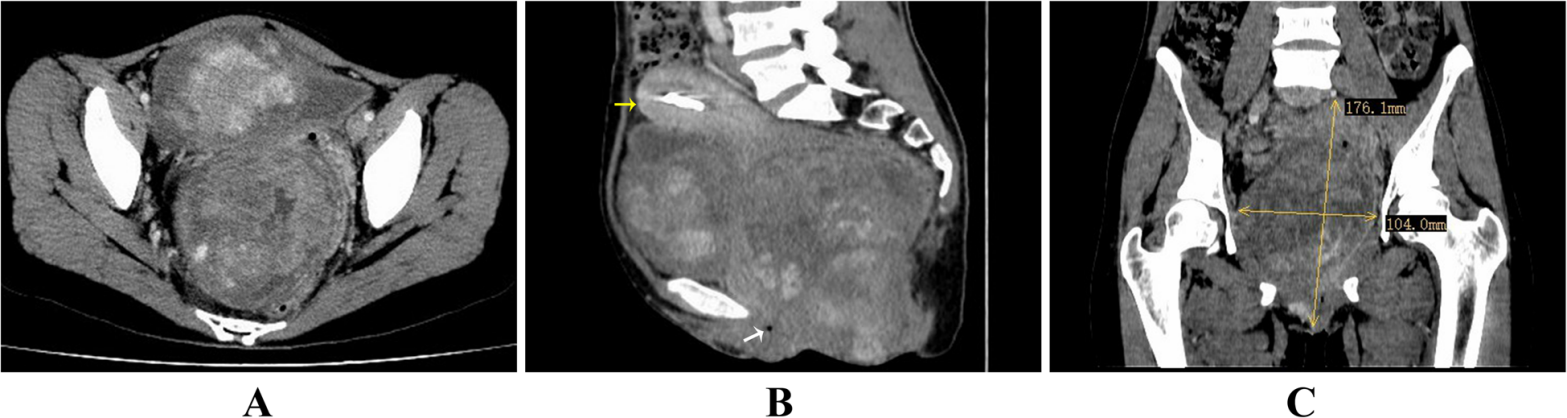
The computed tomography images of the patient with vaginal dedifferentiated liposarcoma. Abdominal computed tomography (CT) showed a 17.6 cm× 10.4 cm solid mass in pelvic cavity. Transverse (**a**), sagittal (**b**) and coronal (**c**) CT images revealed the tumor mass was highly suspected to originate from vagina. The yellow arrow indicate uterus, and the white arrow indicate the part of vaginal space

A 38-year-old female patient, gravid 3 para 2 abortion 1 was referred to our hospital with a one-year history of vaginal mass. One year before admission, the patient felt vaginal uncomfortable and found a mass in her vagina. The patient, lived in a remote rural area, did not go to the hospital for further examination due to her poor economic condition. She experienced discomfort in the vagina and felt the vaginal mass was growing progressively in the past year before admission. Her general medical history revealed no surgery and disease. At the time of consultation, vaginal examination demonstrated a large fixed mass was located in the right lateral wall of the vagina and occupied the upper and middle third of the vagina. Laboratory blood test results, including serum tumor markers, were all within the normal ranges. Abdominal computed tomography (CT) showed a 17.6 cm× 10.4 cm solid mass in pelvic cavity (Fig. 1). The urinary bladder, rectum and uterus were displaced to the left side of the pelvic cavity, but there was no ascites and enlargement of pelvic and para-aortic lymph nodes. Transverse (Fig. 1a), sagittal (Fig. 1b) and coronal (Fig. 1c) CT images revealed the tumor mass was highly suspected to originate from vagina.

Exploratory laparotomy was carried out to remove the mass after adequate preoperative preparation. Intraoperatively, a solid mass with the size of about 18 cm in diameter was found from pelvic floor to pelvic cavity. The lower boundary of the tumor mass was at the level of vaginal orifice. The urinary bladder, rectum, uterus and bilateral adnexa were separate from the mass and displaced to the left side of the pelvic cavity. The boundary between the mass and the surrounding urethra and bladder was clear, and there was no invasion to adjacent organ or metastasis. After Separating the tumor from the surrounding tissue, we found the tumor had a thin, fibrous capsule and originated from the right lateral wall of vagina. The tumor mass was completely removed by blunt dissection.

**Fig. 2:**
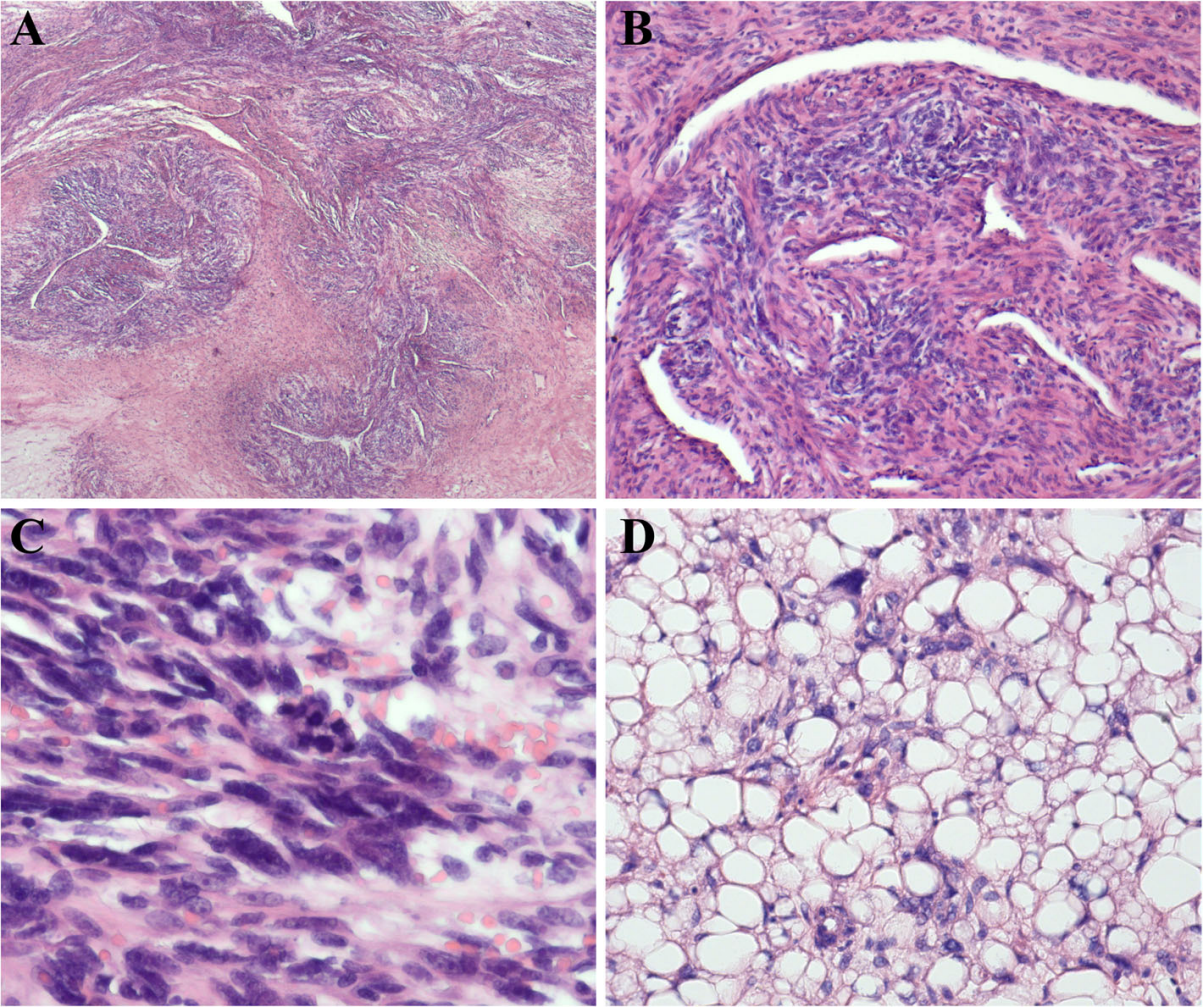
Histologic features of vaginal dedifferentiated liposarcoma. **a**, The tumor was composed of loose fascicles of relatively bland myofibroblast-like cell in fibrous stroma, and resembles fibromatosis. **b**, The cytopathologic features of the tumor showed hypercellularity and infiltrative overgrowth of spindle cells with nuclear atypia. **c**, Mitosis could be seen in the high cellularity areas. The red arrow indicate the mitosis. **d**, Well-differentiated liposarcoma component could be find in the excision specimen, which could contribute to the diagnosis of dedifferentiated liposarcoma

**Fig. 3:**
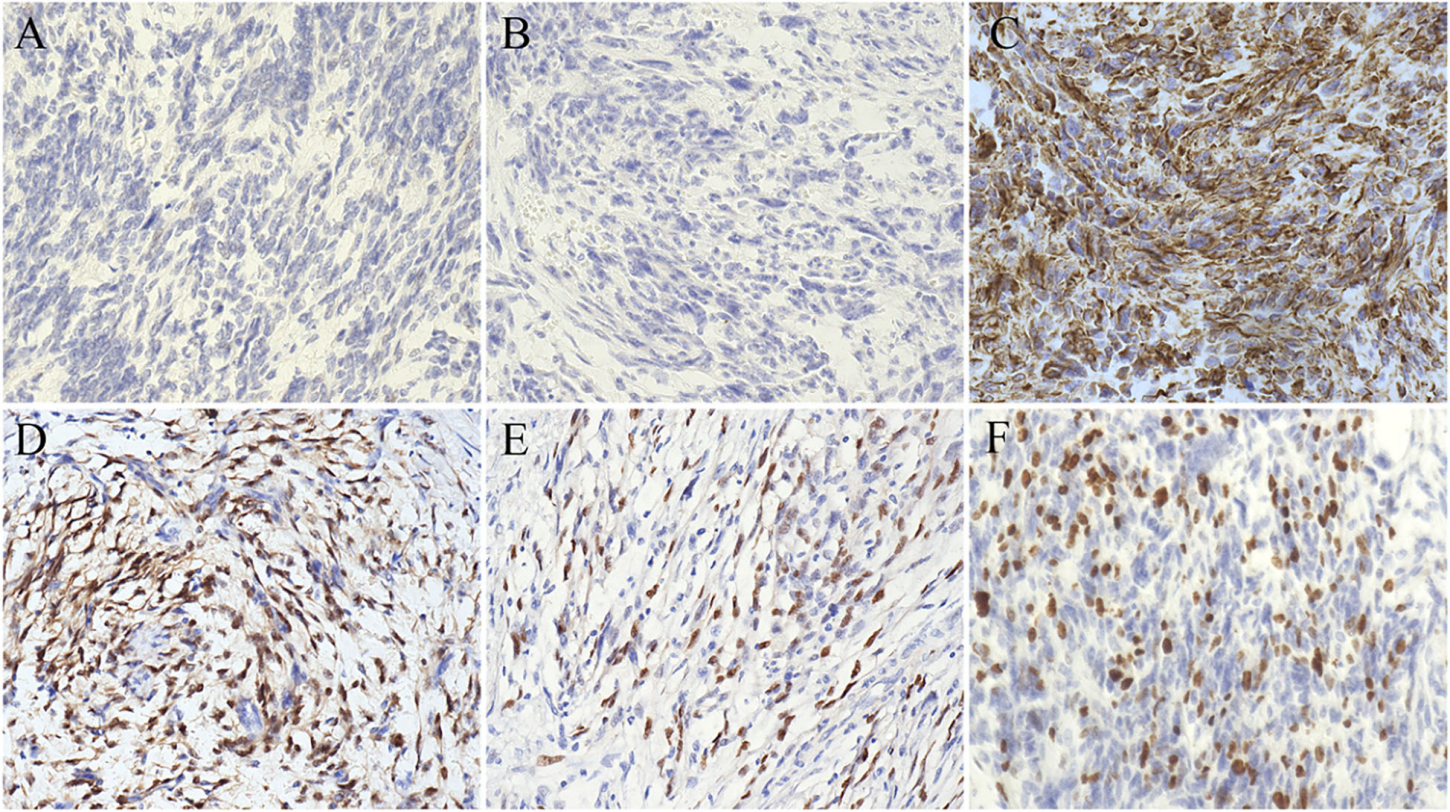
Immunohistochemical staining of vaginal dedifferentiated liposarcoma. Negative expression of Desmin (**a**) and Ck-pan (**b**) was detected, while expression of Vimentin was diffuse and strong in the tumor cell (**c**) using immunohistochemical staining. The combination markers of cyclin-dependent kinase 4 (CDK4) and human murine double minute 2 (MDM2) were useful in the diagnosis of dedifferentiated liposarcoma. Overexpression of CDK4 (**d**) and MDM2 (**e**) were detected. **f**, The positive rate of ki-67 expression was 40–50%. High cellularity areas represent high proliferation

**Fig. 4:**
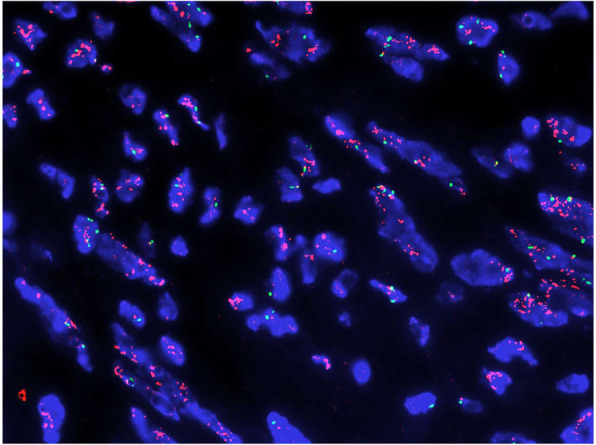
Fluorescence in situ hybridization of vaginal dedifferentiated liposarcoma. The assessment of MDM2 gene amplification by fluorescence in situ hybridization is a highly useful adjunctive diagnostic tool in the diagnosis of dedifferentiated liposarcoma. Red signals represent MDM2, and green signals represent chromosome 12 centromeres

Pathological finding revealed the large mass was multinodular and the cut section was gray-white, with areas of necrosis. Cytopathologic features of the tumor mass showed hypercellularity and infiltrative overgrowth of spindle cells with nuclear atypia (Fig. 2a-c). Histological examination revealed the features of spindle cells with high to moderate cellularity, pleomorphism and moderate to marked cellular atypia with cells disposed in loose fascicles, with a storiform pattern in some parts. The mitotic index was high, and variable amounts of necrosis can be seen (Fig. 2a-c). Its mitotic count was 8 mitoses per 10 high power fields (HPF). In addition, well-differentiated liposarcoma component could be find in the excision specimen (Fig. 2d), which may contribute to the diagnosis of dedifferentiated liposarcoma. The mesthothial tumor was suspected, and then detection of a serial of markers were performed. The tumor lacks epithelial component and cells were rarely stained with p53. These findings didn’t support a diagnosis of anaplastic carcinoma of the vagina. Further, the tumor didn’t express desmin, ck-pan, α-smooth muscle actin, S-100, myoglobin and CD34, suggesting a dedifferentiated tumor (Fig. 3). Overexpressions of human murine double minute 2 (MDM2) and cyclin-dependent kinase 4 (CDK4) were detected. In addition, detection of MDM2 gene amplification was performed by fluorescence in situ hybridization analysis, and the tumor was positive for amplification of the MDM2 gene (Fig. 4). The final histological and immunohistochemical results confirmed the diagnosis of DDLPS deriving from vagina.

The patient was discharged 7 days after surgery without any immediate postoperative complications. Medical oncology was consulted, and additional chemotherapy were recommended. Six courses of combination chemotherapy with a regimen of pirarubicin plus ifosfamide (pirarubicin 30 mg/m^2^, ifosfamide 2000 mg/m^2^) were performed for the patient. The patient remains well with no evidence of disease recurrence with 13 months of follow-up. We are still following-up this patient.

## Discussion

Dedifferentiated liposarcoma (DDLPS), one of the most deadly types of soft tissue sarcoma, is an aggressive and high-grade form of liposarcoma (LPS) [5]. DDLPS is thought to exist on a continuum with well-differentiated liposarcoma (WDLPS), and they together account for 40–45% of all liposarcomas, which comprise 15–20% of all soft tissue sarcomas [4, 5, 9, 10]. DDLPS typically occurs in middle-aged to older adults, most commonly between the ages of 50 and 70 years old [5]. Most of the studies reported that it had no sex predilection, but a recent study including 3573 patients with DDLPS in the National Cancer Database found 65% of cases were male [11]. The total risk of dedifferentiation occurred in WDLPS is nearly 10% [12], while it is significantly higher in WDLPS of the retroperitoneum. Dedifferentiation occurs much less common in the superficial soft tissues, because these being observable sites for which patients are more likely to present earlier with a soft tissue tumor. The most common primary location for DDLPS is the retroperitoneum and extremities, with less frequent sites including head, neck, thorax, and rarely superficially in the soft tissue. Liposarcoma is very rare in female genital tract, and more rare for DDLPS in gynecologic field. There have been very few reports of either retroperitoneal liposarcoma or intra-abdominal liposarcoma in female reproductive system. To the best of our knowledge, there is no reported primary vaginal DDLPS in English and Chinese literature up to date.

DDLPS usually present as slow-growing and painless masses. DDLPS have no specific clinical symptoms, and they are commonly found incidentally. The clinical manifestations usually depend on where the DDLPS occurs. For example, DDLPS occurs in abdominal retroperitoneum may present with symptoms of abdominal pain or distension. DDLPS occurs in vagina may present with vaginal discomfort or pain, like the patient in this study.

Correct diagnosis is extremely difficult before surgical resection. The diagnosis of DDLPS depend entirely on postoperative pathology, but it is reported that a significant percentage of patients with DDLPS had an initial pathological misdiagnosis. Incorrect diagnosis could result in delayed or inadequate treatment. In our case, the patient was initially suspected to have anaplastic vaginal carcinoma because of the rapid extension of the tumor.

Grossly, DDLPS usually present with a large (> 10 cm) and multinodular mass [13], which varies in consistency from medium-firm to solid, soft and myxoid to fleshy. The cut sections of DDLPS may be different and vary from grayish white to tan, or sometimes with areas of necrosis or hemorrhage. The nearby areas of WDLPS may comprise soft yellow, pale, and lobulated fat. The boundary between WDLPS and DDLPS may be abrupt or more subtle. Histologically, DDLPS with components of WDLPS frequently present relatively abrupt boundary between dedifferentiated and well-differentiated areas, but sometimes the transition may be a more gradual, with strands of DDLPS incorporated into the well-differentiated component or a diffuse admixture of dedifferentiated and well-differentiated components. Immunohistochemically, the combination markers of cyclin-dependent kinase 4 (CDK4) and human murine double minute 2 (MDM2) were useful in the diagnosis of dedifferentiated liposarcoma, and there is strong correlation of marker expression with gene amplification status [14]. Use of the combination of CDK4, MDM2 and P16 is helpful in distinguishing DDLPS and WDLPS from other adipocytic tumors in their differential diagnosis. It is now recognized that most neoplasms diagnosed previously as inflammatory malignant fibrous histiocytomas represent DDLPS with these tumors showing 12q13–15 amplifications or gains, as well as immunohistochemical expression of MDM2 and usually CDK4, and amplification of MDM2 and CDK4 with FISH analysis. Vaginal dedifferentiated liposarcoma needs to be differentiated from the soft tissue tumor of the genital tract in clinical differential diagnosis, especially the aggressive angiomyxoma. Histologically, aggressive angiomyxoma is composed of abundant vascularity and spindle cells embedded in an abundant myxoid matrix. Expression of oestrogen and progesterone receptors in the tumour cells are positive. However, FISH analysis for the MDM2 and CDK4 gene amplification is usually helpful in distinguishing aggressive angiomyxoma from dedifferentiated liposarcoma.

Surgery is still the main modality in the treatment of localized DDLPS [15]. Macroscopically wide surgical excision is predictive of improved overall survival in patients with DDLPS [16]. and it has been reported that R2 resection of locally recurrent DDLPS does not improve outcomes when compared with patients who did not receive surgery [17].

Although complete surgical resection is suggested to be standard treatment for DDLPS, neoadjuvant or adjuvant therapy could reduce the risk of recurrence. Systemic therapy combined with surgery can be considered in the treatment of selected patients with localized DDLPS, particularly in patients whose primary tumor is considered borderline resectable or near other anatomically critical structures. However, no prospective reports have shown a survival benefit from neoadjuvant or adjuvant chemotherapy in patients with DDLPS [17].

Similar to other DDLPS, the first-line chemotherapy regimens for vaginal DDLPS are still base on anthracycline, and the common chemotherapy regimen is single-agent anthracycline or anthracycline in combination with ifosfamide. Ifosfamide, an alkylating agent, is typically combined with pirarubicin in the first-line setting in order to maximize benefit. In the present case, the patient underwent complete surgical resection for the mass. Six courses of a combination chemotherapy of pirarubicin and ifosfamide were administered to the patient, and the patient remains well with no evidence of disease recurrence with 13 months of follow-up, indicating that combination chemotherapy with pirarubicin and ifosfamide may be effective for treating vaginal DDLPS.

Although anthracycline-based chemotherapy remains the standard treatment for selected patients with DDLPS after surgery, recent study has shown a number of systemic agents have clinical benefit for patients with metastatic or unresectable DDLPS [5]. The optimal treatment methods for DDLPS patients should depend on the following factors including extent of disease, comorbid conditions, symptoms and performance status. Therefore, the decision for adjuvant or neoadjuvant chemotherapy should be made on a case-by-case basis. Recently, the checkpoint inhibitors including MDM2 antagonists and CDK4 antagonists have been shown to have some therapeutic effects in the treatment of DDLPS, and future clinical studies will help to establish novel therapeutic strategies for DDLPS.

## Conclusion

Dedifferentiated liposarcoma is an aggressive and high-grade form of liposarcoma. Liposarcoma is very rare in female genital tract, and more rare for DDLPS in gynecologic field. Correct diagnosis is extremely difficult before surgical resection. The diagnosis of DDLPS depend entirely on postoperative pathology. We have presented, to the best of our knowledge, the first case of DDLPS deriving from the vagina. The patient remains well with no evidence of disease recurrence after complete surgical resection followed by six courses of systemic chemotherapy, indicating that combination chemotherapy with pirarubicin and ifosfamide may be effective for treating vaginal DDLPS. Novel therapeutic strategies are needed for better outcomes in patients with DDLPS.

## Data Availability

Not applicable.

## Abbreviations

LPS: Liposarcoma
DDLPS: Dedifferentiated liposarcoma
WDLPS: Well-differentiated liposarcoma
